# A Highly Sensitive “on-off” Time-Resolved Phosphorescence Sensor Based on Aptamer Functionalized Magnetite Nanoparticles for Cadmium Detection in Food Samples

**DOI:** 10.3390/foods9121758

**Published:** 2020-11-27

**Authors:** Bin Lai, Ruiying Wang, Xiaoting Yu, Haitao Wang, Zhouping Wang, Mingqian Tan

**Affiliations:** 1 School of Food Science and Technology, Dalian Polytechnic University, Qinggongyuan 1, Ganjingzi District, Dalian 116034, China; bin.lai33@gmail.com (B.L.); wangruiying2020_0@163.com (R.W.); xiaotingddpp12@163.com (X.Y.); wanght@dlpu.edu.cn (H.W.); 2 National Engineering Research Center of Seafood, Dalian Polytechnic University, Dalian 116034, China; 3 Collaborative Innovation Center of Seafood Deep Processing, Dalian Polytechnic University, Dalian 116034, China; 4 School of Food Science and Technology, Jiangnan University, Wuxi 214122, China; wangzp@jiangnan.edu.cn

**Keywords:** aptamer, cadmium, magnetic separation, time-resolved luminescence

## Abstract

Cadmium contamination is a severe threat to food safety. Therefore, the development of sensitive and selective cadmium detection strategies is urgently required. The elimination of background autofluorescence generated from the food matrix is critical to the optical assay for cadmium detection. Herein, a time-resolved phosphorescence sensor based on an “on-off” strategy was developed for cadmium determination in food samples. The phosphorescence nanoparticles were used as a luminous material to minimize the interference of background autofluorescence. The cadmium-binding aptamer was immobilized onto the magnetic beads and combined with a black hole quencher 1 (BHQ_1_) with complementary DNA as the target recognition element. With the presence of cadmium, the cadmium-binding aptamer bound to cadmium specifically and resulted in the release of BHQ_1_. The free BHQ_1_ remained in the solution after magnetic separation and quenched the phosphorescence. The phosphorescence intensity was negatively related to the concentration of cadmium. Under optimal conditions, the time-resolved phosphorescence sensor showed a linear response to cadmium concentration within a range from 0.05 to 5 ng mL^−1^ and with a detection limit of 0.04 ng mL^−1^. This “on-off” time-resolved phosphorescence sensor was successfully applied for cadmium detection in spring water and clam samples, which provided a rapid and straightforward method.

## 1. Introduction

Cadmium (Cd^2+^) is one of the most hazardous heavy metal elements [1] and shows toxicity to multi-organs, such as heart, brain, liver, bone, and kidney, even in a low concentration [2]. According to the recommendation of the World Health Organization (WHO), the contamination of cadmium in drinking water should no more than 3 ng mL^−1^ [3]. Therefore, a sensitive and rapid quantification method for cadmium is highly demanded.

Optical detection sensors based on aptamer have been widely applied in various areas due to their highly selective and economic efficiency, and these areas include environmental measurement [4], clinical diagnosis [5], and food safety [6]. Recently, various strategies based on aptamer for metal ions detection have been developed, including Hg^2+^ detection using DNA-modified gold sensor, Ag^+^ detection using graphene nanoprobe, and Pb^2+^ detection using graphene quantum dots [7,8,9]. For example, Tao et al. [3] used gold nanoparticle modified MoS_2_ nanocomposites as an enzyme mimic to enhanced peroxidase-like activity and improved the sensibility of colorimetric Cd^2+^ detection. Zhou et al. [10] designed a G-rich sequence for the aptamer to bind with Cd^2+^ and form a G-quadruplex structure as a peroxidase-like DNAzyme to enhance the catalytic activity of heme in order to provide a fluorescent signal for cadmium detection. Zhu et al. [11] decorated Carboxyfluorescein(FAM)-labeled 3′ end and poly-G structure 5′ end to the aptamer and its conformation changed from a random coil sequence to a stem-loop structure when it met Cd^2+^ and this resulted in the fluorescence of FAM change which linked with Cd^2+^ concentration. However, some drawbacks still existed in these reported methods, such as multiple centrifugal procedures being required, labelling the aptamer with the exogenous marker and the long incubation time for testing. Therefore, it is useful to develop a simple, sensitive, easy-to-use sensor for cadmium detection in complex samples.

The major challenges for fluorometric sensors are the autofluorescence that originates from the matrix. The luminophores used in these sensors, such as organic dyes, quantum dots, and up-conversion nanoparticles [12], are easily interfaced by autofluorescence since the fluorescence spectrophotometer cannot distinguish the fluorescence signal emitted by these elements from the matrix [13]. Instead, the phosphorescence from long-lived nanoparticles can be distinguished from the autofluorescence using the time-resolved techniques, due to their different lifetime. Thus, the autofluorescence interference from the matrix can be eliminated by introducing phosphorescence materials as the signal elements in a detection system. To this end, long-life time Zn_2_GeO_4_:Mn nanorods have been applied in time-resolved assay in complex samples [14]. For instance, Wang et al. [15] functionalized Zn_2_GeO_4_:Mn with a lysozyme-binding aptamer for lysozyme detection in serum. Shi et al. [16] detected prostate-specific antigens and carcinoembryonic antigens by using a Zn_2_GeO_4_:Mn and Zn_2_GeO_4_:Cr modified aptasensor. Therefore, the application of long-lived photoluminescence nanomaterials in cadmium detection is feasible and actionable by the time-resolved technique.

**Scheme 1:**
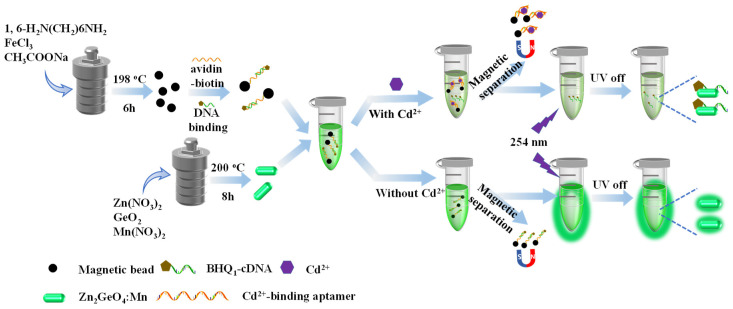
Scheme illustration of the “on-off” time-resolved sensor based on magnetic separation for Cd^2+^ detection.

In this study, an “on-off” time-resolved aptamer sensor was developed based on magnetic separation for rapid and sensitive detection of cadmium in food samples. The detection principle of this developed aptasensor is shown in Scheme 1. Cd^2+^-binding aptamers bound to magnetic beads through biotin-avidin mediated conjugation. After hybridization with the black hole quencher 1 (BHQ_1_)-modified complementary DNAs (cDNAs), the magnetic beads acted as cadmium recognition moiety. Furthermore, the time-resolved phosphorescence Zn_2_GeO_4_:Mn was used as the luminous signal emitter. At the presence of Cd^2+^, the BHQ_1_-cDNAs would fall off from the magnetic beads since the binding affinity of Cd^2+^-binding aptamers against cadmium was higher than BHQ_1_-cDNAs. Under an external magnetic field, the magnetic beads combined with Cd^2+^ were removed, and the released BHQ_1_-cDNAs were still in solution, which would bind to the Zn_2_GeO_4_:Mn nanorods and quench the phosphorescence through the phosphorescence resonance energy transfer (PRET) mechanism. The phosphorescence of the long-live Zn_2_GeO_4_:Mn changed from “on” to “off” with the introduction of cadmium. The intensity of the phosphorescence signal was closely related to the concentration of cadmium and thus achieved cadmium quantification. Moreover, the testing sample can be prepared and tested without the washing and centrifuge step due to the introduction of magnetic separation. Importantly, the application of long-lived phosphorescence materials improved the ratio of signal to noise, thus increasing the sensitivity of the sensor in complex samples too.

## 2. Materials and Methods

### 2.1. Materials and Instrumentation

Germanium oxide (GeO_2_, 99.999%), glutaraldehyde (25% in H_2_O), ferric chloride (FeCl_3_·6H_2_O), 1,6-hexamethylenediamine, sodium acetate, potassium chloride, sodium chloride, disodium phosphate (Na_2_HPO_4_·12H_2_O), and dipotassium phosphate (K_2_HPO_4_) were purchased from Sinopharm Chemical Reagent Co., Ltd. (Shanghai, China). Zinc nitrate hexahydrate (Zn(NO_3_)_2_·6H_2_O, 99.998%), manganese (II) nitrate hydrate (Mn(NO_3_)_2_·4H_2_O, 98%), urea, avidin, nitric acid, hydrogen peroxide solution, (3-aminopropyl) triethoxysilane (APTES), N, N-dimethylformamide (DMF), and standard solution of Cd^2+^ (1000 μg mL^−1^ in 1.0 mol L^−1^ HNO_3_) were purchased from Shanghai Aladdin Bio-Chem Technology Co., Ltd. (Shanghai, China). All reagents were at least of analytical purity and without further purification. The water used in this study was ultrapure water from a Milli-Q Integral water purification system (Millipore, Bedford, MA, USA). The aptamer and its complementary strand (Table S1) were chosen according to the previous study [17] and synthesized from Sangon Biotechnology Co., Ltd. (Shanghai, China) with PAGE purification.

### 2.2. Characterization

Transmission electron microscope (TEM) images were taken through the JEM-2100 (JEOL, Tokyo, Japan). The crystal phase of nanorods was measured through an X-ray diffraction (XRD) instrument (XRD-6100, Shimadzu, Kyoto, Japan). FT-IR spectra were performed on a Fourier transforms infrared spectrometer (Perkin Elmer, Norwalk, CT, USA) via the KBr tablet. X-ray photoelectron spectroscopy (XPS) spectra were taken from an ESCALAB 250 X-ray photoelectron spectrometer (Thermo VG, Waltham, WA, USA) for chemical composition. For element analysis, energy dispersive spectroscopy (EDS) was conducted by using the EDS analyzer (X-Max50, Oxford Instruments Co., Oxford, UK). A UV-2300 spectrometer (Shimadzu, Japan) was used to collect Ultraviolet-visible (UV-vis) spectra at room temperature. The concentration of aptamers and complementary strands were measured spectrophotometrically with NanoDrop (NanoDrop One, Thermo Fisher Scientific, Waltham, MA, USA). The photoluminescence and the time-resolved luminescence decay spectra were determined by FLS980 spectrometer (Edinburgh Instruments Co., Livingston, UK). The detections of Cd^2+^ were measured by using a multi-function microplate reader (Synergy H1, BioTek Instruments, Inc., Winooski, VT, USA).

### 2.3. Synthesis of Phosphorescence Nanorods

Zn_2_GeO_4_:Mn nanorods were synthesized using hydrothermal methods for 2, 4, 6, and 8 h, respectively, according to the reported methods with some modifications [15,18]. Briefly, 2.0 mmol zinc nitrate hexahydrate, 0.005 mmol manganese (II) nitrate hydrate, 1 mmol germanium oxide were dissolved in 300 μL nitric acid and diluted with 11 mL deionized water. After adding urea to adjust pH to 9.5, the reaction mixture was vigorously stirred at room temperature for 1 h. The mixed solution was transferred to a Tefon-lined stainless autoclave and kept at 200 °C for different time periods (2, 4, 6, 8 h) to obtain different samples. After reaction, the mixture was cooled down to room temperature, the products were centrifugated, then washed three times with deionized water and ethanol. The quantum yields (QY) of the Zn_2_GeO_4_:Mn nanorods were measured using quinine sulfate (0.1 M H_2_SO_4_, quantum yield 54%) as a reference [19].

### 2.4. Synthesis of Amine-Functionalized Magnetic Beads

The amine-functionalized magnetic beads were hydrothermally synthesized following the previous method [20]. Briefly, 1.0 g ferric chloride and 2.0 g sodium acetate were dissolved in 30 mL ethanediol. A total of 6.5 g 1,6-hexamethylenediamine was added under vigorous stirring at 50 °C. The claret-red solution was then transferred into a Teflon-lined autoclave and heated at 198 °C for 6 h. After cooling down to room temperature, the amine-functionalized magnetic beads were separated by the external magnetic field and washed with ethanol and water three times.

### 2.5. Immobilization of Cd^2+^-Binding Aptamers onto Magnetic Beads and Complementary DNA Hybridization

The amine-functionalized magnetic beads were coated with avidin before binding with biotinylated Cd^2+^-binding aptamers. In detail, 5 mg amine-functionalized magnetic beads were activated with 25% glutaraldehyde. Then the mixture was incubated with avidin (1 mg mL^−1^) in PBS buffer (pH = 7.4) for 12 h. Subsequently, avidin coated magnetic beads were collected through magnetic separation and washed to remove the unbound avidin. The biotin-labelled aptamer solution (20 μL, 0.1 μM) was incubated with avidin-coated magnetic beads for 1 h at 37 °C. The aptamer-magnetic beads were separated magnetically and mixed with 40 μL BHQ1-cDNA (0.1 μM) in 500 μL volume. Then the aptamer and complementary chain were annealed for 10 min at 95 °C, followed by cooling down in ice for 10 min and incubation at 37 °C. One hour later, the probes were separated using a magnet and stored at 500 μL PBS for further use.

### 2.6. Detection of Cd^2+^

The Cd^2+^ detection curve was established using 100 μL of BHQ_1_-aptamer-magnetic beads target probes, 100 μL of Zn_2_GeO_4_:Mn phosphorescent probes and 300 μL of samples of different concentrations of Cd^2+^ (0.05, 0.5, 1.0, 1.5, 3.5, 5.0, 10.0, 20.0, 30.0, 40.0, and 50.0 ng mL^−1^). The mixture was incubated for 15 min at 37 °C, and then the Cd^2+^ probes were magnetically separated to the bottom. The phosphorescence intensity of the supernatant sample was measured at 540 nm for 1500 μs with a multi-function microplate reader (BioTek Instruments, Inc., Winooski, VT, USA) with a gating time of 100 μs to eliminate the auto-fluorescence from background matrix.

### 2.7. Selectivity of Cd^2+^

Selectivity is the essential factor for a sensor, and the selectivity of the aptasensor against other metal ions was investigated. In detail, 30 ng mL^−1^ of As^3+^, Hg^2+^, Mn^2+^, Al^3+^, Mg^2+^, Pb^2+^, Zn^2+^, K^+^, Ag^+^, Fe^2+^, Cu^2+^ and 5 ng mL^−1^ Cd^2+^ were mixed with BHQ_1_-aptamer-magnetic beads target probes and Zn_2_GeO_4_:Mn phosphorescent nanorods, respectively. After the mixture was incubated for 15 min, the magnetic probes were removed with the external magnetic field, and the phosphorescence intensity of these supernatants was measured with 100 µs gate time. All tests were repeated three times in parallel.

### 2.8. Food Sample Preparation

The spiked recovery experiments were performed to evaluate the accuracy of the developed aptasensor. Green willow clams (*Mactra chinensis*) meat and spring water were purchased at a local market (Dalian, China) and spiked with Cd^2+^ for detection. The pressure digestion method was used for sample treatment. The clam samples were washed thoroughly with deionized water to remove the silts, and the prewashed clam meats were homogenized. Then the homogenized clam meat was spiked with different amounts of cadmium (0.025, 0.087, 0.125 mg kg^−1^ Cd^2+^) and thoroughly mixed. A total of 1.0 g homogenized Cd^2+^ spiked clam sample was weighted from different mixtures and digested with 5 mL of HNO_3_ in polytetrafluoroethylene vessel overnight, respectively. After further adding 2 mL H_2_O_2_ (30%), the vessels were tightly capped and kept at 160 °C for 6 h until clear solution was obtained. After the solvent had evaporated, the digestion sample pH was adjusted to 7.5 with sodium hydroxide solution and diluted with PBS buffer (pH = 7.5) to a total volume of 25 mL for further detection. Spring water was filtered with a 0.22 μm filter membrane before Cd^2+^ measurement.

### 2.9. Statistical Analysis

All measurements in this experiment were done three times. Results were presented as mean values ± standard error of the mean (SEM). The software Orgin Pro 2018 (Microcal, Northampton, MA, USA) was used for plotting graphs in the figures.

## 3. Results and Discussion

### 3.1. Characterization of Zn_2_GeO_4_:Mn Nanorods

**Figure 1:**
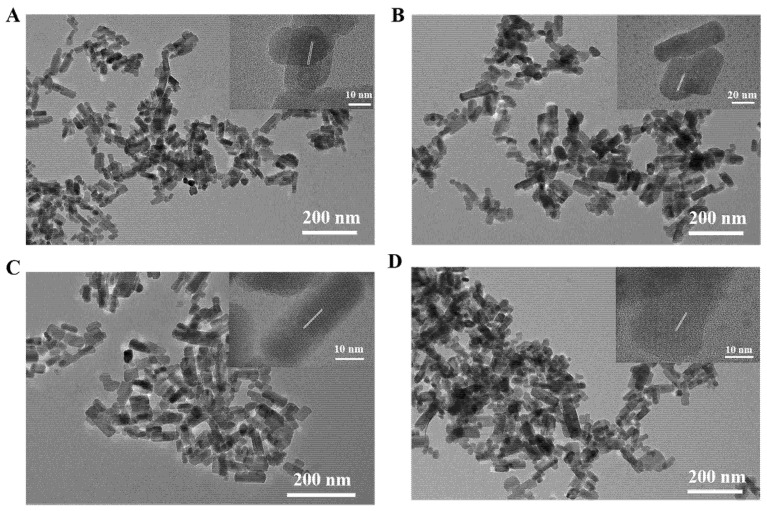
Transmission electron microscope (TEM) and high resolution (HR)TEM (insets) images of Zn_2_GeO_4_:Mn nanorods synthesized in 2 h (**A**), 4 h (**B**), 6 h (**C**), 8 h (**D**).

The Zn_2_GeO_4_:Mn nanorods were hydrothermally synthesized at 200 °C for different reaction time periods (2, 4, 6, 8 h). TEM images indicated that the prepared Zn_2_GeO_4_:Mn nanorods had rod-shaped morphology with clear lattice fringes, which exhibited good water dispersity (Figure 1A–D). Along with increasing reaction times, the average length of nanorods increased slightly from 45 to 65 nm (Figure S1A–D), and the average width increased gradually from 17 to 25 nm (Figure S1E–H). This demonstrated that the longer reaction time, the bigger the Zn_2_GeO_4_:Mn nanorods. The reason why different reaction times were used was that the Zn_2_GeO_4_:Mn nanorods with an appropriate size and better optical properties were desired for Cd^2+^ detection.

**Figure 2:**
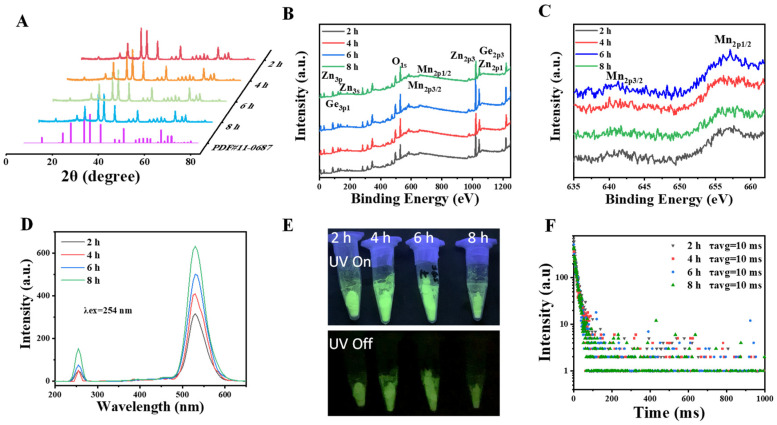
(**A**) X-ray diffraction (XRD) pattern, (**B**) X-ray photoelectron spectroscopy (XPS) survey, (**C**) high-resolution XPS spectra of Mn^2+^, (**D**) phosphorescence emission spectra of Zn_2_GeO_4_:Mn nanorods synthesized in 2, 4, 6, 8 h. (**E**) Photograph of Zn_2_GeO_4_:Mn nanorods excited by 254 nm UV light and its persistent luminescence after turning off the UV light. (**F**) The effects of reaction times on phosphorescence lifetime of Zn_2_GeO_4_:Mn.

The microcrystalline structure of the Zn_2_GeO_4_:Mn nanorods were further analyzed by X-ray diffraction. The XRD results indicated that manganese doping showed a neglected effect on the trigonal crystal system of the Zn_2_GeO_4_ nanorods, which were consistent with the standard card PDF#11-0687 (Figure 2A). The bonding configuration and composition of Zn_2_GeO_4_:Mn nanorods were analyzed by EDS elemental mapping and X-ray photoelectron spectroscopy. The EDS results (Figure S2A–D) proved that these nanorods contained elements of zinc, germanium, oxygen, and manganese. The XPS spectra of Zn_2_GeO_4_:Mn nanorods in Figure 2B show that the characteristic binding energy values of Zn, Ge, Mn, and O element peaks are observed, which are consistent with the EDS results. The high-resolution XPS spectra of Zn _2p_ can be fitted into two peaks at around 1023 and 1046 eV, which corresponds to the 2p_3/2_ and 2p_1/2_ core levels, respectively. The energy difference between the two peaks could confirm the presence of zinc ions of the divalent form [21]. The XPS peak (Figure S3A–D) at around 1222 eV corresponds to the 2p3 peaks of Ge. Meanwhile, the XPS peak of the O_1s_ was observed at 531 eV (Figure S3E–H). The Mn_2p_ XPS spectra in Figure 2C indicate that the Mn 2p_3/2_, and Mn 2p_1/2_ spin-orbit peaks are displayed at 642 and 653 eV, respectively. The manganese (II) nitrate hydrate was used as the manganese source for the synthesis, and the valence of Mn in the Zn_2_GeO_4_:Mn nanorods remained unchanged [22]. Both EDS and XPS results confirmed that the Mn^2+^ ions were successfully doped in Zn_2_GeO_4_ nanorods.

The emission spectra of Zn_2_GeO_4_:Mn nanorods in Figure 2D show the maximum emission peaks at 540 nm with 254 nm excitation for all the Zn_2_GeO_4_:Mn nanorods. Notably, the luminescence intensities of these nanorods were positively correlated with reaction time. Meanwhile, the persistent green luminescence of Zn_2_GeO_4_:Mn nanorods can be observed after turning off the excitation (Figure 2E). The green emission of Zn_2_GeO_4_:Mn nanorods could be ascribed to the transition of Mn^2+^ from the excited state of ^4^T_1_(^4^G) to the ground state of ^6^A_1_(^6^S) [23]. These results were consistent with the EDS and XPS results and proved the successful doping of Mn^2+^. The phosphorescence lifetimes of Zn_2_GeO_4_:Mn nanorods were also measured. As shown in Figure 2F, the reaction time have limited effects on phosphorescence lifetime. Furthermore, the effect of reaction time on the quantum yields of Zn_2_GeO_4_:Mn nanorods were investigated. The quantum yields of Zn_2_GeO_4_:Mn nanorods increased from 8.42% to 17.82% with the extension of reaction time (Table S2). These results were aligned with the emission spectra of Zn_2_GeO_4_:Mn nanorods. These results confirmed that the Zn_2_GeO_4_:Mn nanorods with long-lived lifetime were successfully synthesised using the facile hydrothermal method. In this study, the Zn_2_GeO_4_:Mn nanorods synthesized for 8 h were selected as signal elements due to their high quantum yield.

### 3.2. Characterization of Magnetic Probes for Cd^2+^ Detection

**Figure 3:**
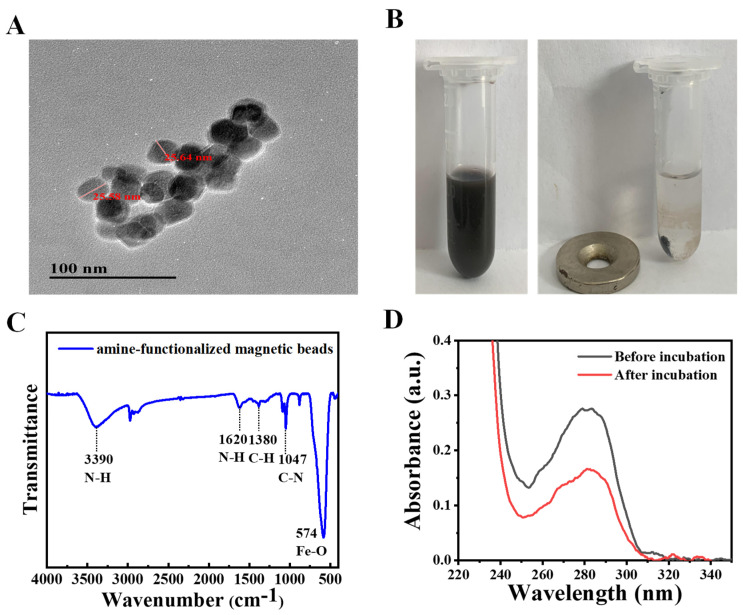
(**A**) Transmission electron microscope (TEM) image of amine-functionalized magnetic beads. (**B**) Photograph of amine-functionalized magnetic beads in aqueous solution (magnet absence and presence). (**C**) Fourier transform infrared (FT-IR) spectra of amine-functionalized magnetic beads. (**D**) Ultraviolet-visible (UV-vis) spectra of biotin-modified aptamer solution before and after incubation with avidin-functionalized magnetic beads.

The size distribution and morphology of magnetic beads were investigated through TEM images. The results show that the magnetic beads are spherical in size and shape, with an average diameter of 25.6 nm (Figure 3A). The magnetic beads could be dispersed in an aqueous solution after sonication and aggregated under external magnetic fields (Figure 3B). This proved that the magnetic beads were successfully synthesized and had a good magnetic property for magnetic separation. FT-IR spectra confirmed the presence of amino groups on the surface of magnetic beads. As shown in Figure 3C, the strong bond at 574 cm^−1^ was assigned to Fe-O vibrations [24]. The bond at 1047 cm^−1^ was attributed to C-N stretching vibration and the bond at 1380 cm^−1^ was due to C-H vibration. Furthermore, the bonds at 1620 and 3390 cm^−1^ were assigned to N-H stretches [25]. The FT-IR results consisted of the previous observation [25] and demonstrated that the magnetic beads had been successfully modified with amino groups.

The amino group of magnetic beads could be conjugated with bio-active molecules by glutaraldehyde via Schiff base reaction. Thus, glutaraldehyde was applied to induce the cross-linking of amine-functionalized magnetic beads and avidin. As shown in Figure 3D, the avidin had a significant absorption peak at a wavelength of 280 nm. After incubation with the amine-functionalized magnetic beads, the absorption peak of avidin in the supernatant was decreased to half. The decrease of UV absorption indicated that the avidin molecules were coupled with magnetic beads. Moreover, the avidin-functionalized magnetic beads were then reacted with biotinylated Cd^2+^-binding aptamers.

### 3.3. Optimization of Experimental Conditions for the Cd^2+^ Detection

**Figure 4:**
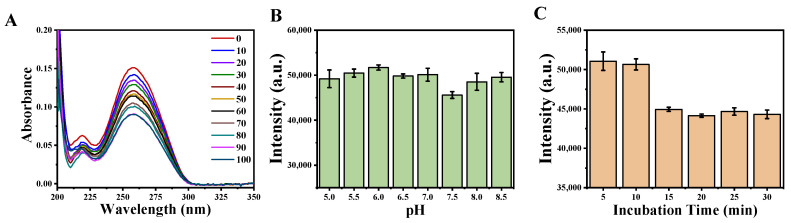
Optimization assay conditions: (**A**) Ultraviolet-visible (UV-vis) spectra of the various mass ratio of biotinylated aptamer and avidin-coated magnetic beads. (**B**) Phosphorescence intensity of the sensor upon detection of Cd^2+^ at different pH conditions. (**C**) Phosphorescence intensity at various incubation times for sensor upon detection of Cd^2+^.

In order to obtain a high sensitivity of Cd^2+^ detection, the experimental conditions were optimized by reacting Cd^2+^-binding aptamers with different mass ratio of avidin-coated magnetic beads from 1:0 to 1:100 (1:0 is the pure aptamer as reference). First, the amounts of Cd^2+^-binding aptamers conjugated onto avidin-coated magnetic beads were determined by UV absorption of aptamer at 260 nm. As demonstrated in Figure 4A, the UV absorption spectra at 260 nm of aptamers were continually decreased with the increase of avidin-coated magnetic beads, until the mass ratio reached 1:90. This result suggested that the maximum usage of avidin-coated magnetic beads had been reached. Thus the optimized mass ratio of Cd^2+^-binding aptamers and avidin-coated magnetic beads in this sensor was 1:90. Second, 5 μg L^−1^ of Cd^2+^ was used as the sample to determine the effect of pH on the detection process. The phosphorescence intensity at different pH solution from 5.0 to 8.5 were recorded (Figure 4B). The results showed that the phosphorescence intensity of the sensor decreased slightly and reached its minimum value when the pH was 7.5. Therefore, the pH of the reaction buffers was adjusted to 7.5 as the optimum pH value. Third, the reaction time was optimized since it was critical to detection efficiency. As shown in Figure 4C, the phosphorescence intensity decreased with the increase of reaction time. After 15 min, the phosphorescence intensity became stable. Therefore, 15 min of reaction time was considered as the optimized reaction time for this detection method.

### 3.4. Quantification of the Cd^2+^ Assay

**Figure 5:**
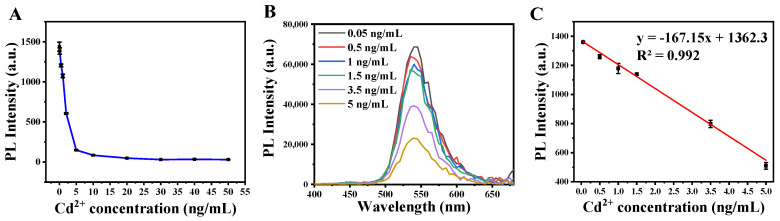
(**A**) Phosphorescence intensity versus Cd^2+^ concentration. (**B**) The phosphorescence spectra of the presence of Cd^2+^ range from 0.05 to 5 ng mL^−1^. (**C**) The linear calibration of the phosphorescence intensity against Cd^2+^ concentration.

This aptasensor was then applied to detect Cd^2+^ under the optimized conditions. The phosphorescence intensity decreased with the increase of Cd^2+^ concentration (Figure 5A). The phosphorescence spectra of this aptasensor are shown in Figure 5B in various concentrations of Cd^2+^. The detection probe combined with Cd^2+^ was removed by magnetic separation. The BHQ_1_-cDNAs were released from detection probes and remained in supernatant solution. As reported previously, the electrostatic repulsion between the phosphate backbone and Zn_2_GeO_4_:Mn was lower than the coordination interaction between nitrogen atoms of unfolded ssDNA and the Zn_2_GeO_4_:Mn [26]. Thus the cDNAs connected with Zn_2_GeO_4_:Mn through coordination interaction and BHQ_1_ quenched the phosphorescence of Zn_2_GeO_4_:Mn. The phosphorescence intensity showed a linear relationship with Cd^2+^ concentration (Figure 5C), in the range from 0.05 to 5 ng mL^−1^, with the equation of y = −167.15x + 1362.3 (R^2^ = 0.992), where y is the phosphorescence intensity, and x is Cd^2+^ concentration. The limit of detection (LOD) of the calibration curve was 0.04 ng mL^−1^ (0.52 nM), which was calculated based on 3σ/S (σ was the standard deviation of the blank signal (*n* = 9), and S was the slope of the fitted curve).

### 3.5. Selectivity Test and Cd^2+^ Assay in Real Food Samples

**Figure 6:**
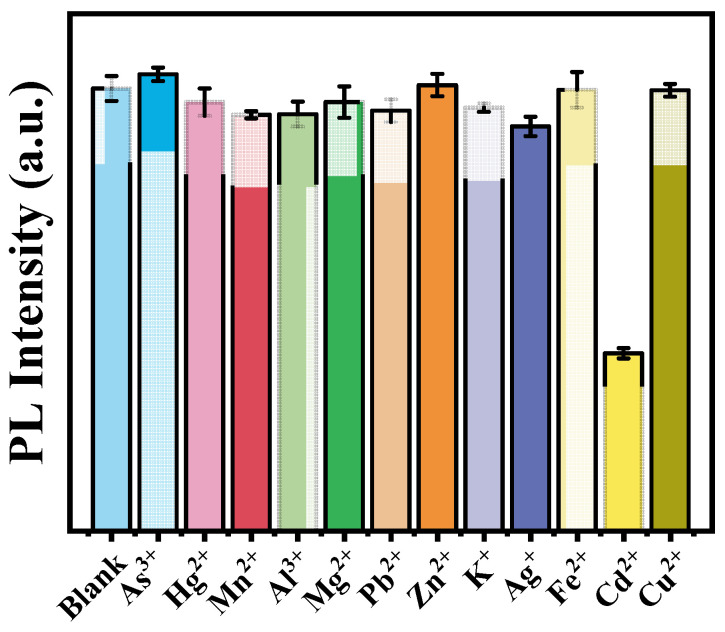
The selectivity of the aptasensor for Cd^2+^ against As^3+^, Hg^2+^, Mn^2+^, Al^3+^, Mg^2+^, Pb^2+^, Zn^2+^, K^+^, Ag^+^, Fe^2+^, and Cu^2+^.

Selectivity is the essential factor for a sensor, and the selectivity of the prepared aptasensor against other metal ions was also evaluated. Under the detecting condition, 30 ng mL^−1^ of As^3+^, Hg^2+^, Mn^2+^, Al^3+^, Mg^2+^, Pb^2+^, Zn^2+^, K^+^, Ag^+^, Fe^2+^, Cu^2+^ and 5 ng mL^−1^ Cd^2+^ were mixed with BHQ_1_-aptamer-magnetic beads target probes and Zn_2_GeO_4_:Mn phosphorescent signal element, respectively. As shown in Figure 6, the phosphorescence intensities were almost unaffected by these interference metal ions. The significant decrease in phosphorescence intensity can be obvious only in the Cd^2+^ solution. This result confirmed that the developed sensor was highly specific to Cd^2+^.

Table 1 shows the results of Cd^2+^ detection in spring water and surf clam by the aptasensor. The spring water samples were spiked with 1, 3, 5 ng mL^−1^ Cd^2+^ and the clam meats were mixed with 0.025, 0.087, and 0.125 mg kg^−1^ Cd^2+^. The quantitative analysis of Cd^2+^ were 1.08 ± 0.08, 3.36 ± 0.03, 5.45 ± 0.16 ng mL^−1^ for spring water and 0.027 ± 0.002, 0.089 ± 0.002, 0.135 ± 0.002 mg kg^−1^ for surf clam, respectively. The recovery range of this method was from 101.41% to 112%, with the relative standard deviations 0.08–8.04%. These results indicated that the “on-off” time-resolved phosphorescence sensor had good measurement precision and might have potential in Cd^2+^ detection in complex food samples.

The comparison of the limit of detection (LOD) of this aptasensor with other methods is listed in Table 2. The LOD of this method was not only lower than that of the colourimetric methods using Au nanoparticles (NPs) or Au-MoS2 nanocomposites but also lower than that of the fluorescence method by SYBR^®^ green I or carboxyfluorescein (FAM). These could be attributed to the use of phosphorescence signals to overcome the interference of sample autofluorescence from the background, and the magnetic detection probes were ready to be collected by the external magnetic field, thus simplifying the test process. The aptasensor had excellent signal to noise ratio, good selectivity of Cd^2+^ and easy operation steps due to the introduction of a magnetic separation strategy. In particular, the detection of Cd^2+^ in surf clam demonstrated that the aptasensor was more applicable for real sample detection.

## 4. Conclusions

A highly sensitive “on-off” time-resolved phosphorescence sensor based on aptamer functionalized magnetite nanoparticles was developed for Cd^2+^ detection in spring water and surf clams. The sensor was designed based on the Cd^2+^ competitive binding to the aptamer, causing the release of BHQ_1_-cDNAs to quench phosphorescence in order to realize “on-off”. The autofluorescence interference was eliminated using the time-resolved technique because the long-lived lifetime of the Zn_2_GeO_4_:Mn allowed light emission to persist for enough time to eliminate autofluorescence from the samples. The photoluminescence sensor had a good linear response from 0.05 to 5 ng mL^−1^ with a LOD of 0.04 ng mL^−1^. The magnetic separation function of the aptasensor enabled the facile and fast testing of the Cd^2+^. Further studies for the detection of food samples without pretreatment are desired, and this “on-off” time-resolved phosphorescence sensor based on aptamer functionalized magnetite nanoparticles is promising for the development of cadmium detection in food samples.

## Figures and Tables

**Table 1:** Recovery ratio of Cd^2+^ in spring water and surf clam samples.

Sample	Added	Detected (Mean *n* = 3)	Recovery (%)
Spring water	1 ng mL^−1^	1.08 ± 0.08 ng mL^−1^	108.46 ± 8.04
	3 ng mL^−1^	3.36 ± 0.03 ng mL^−1^	112.00 ± 0.98
	5 ng mL^−1^	5.45± 0.16 ng mL^−1^	109.00 ± 3.16
surf clam	0.025 mg kg^−1^	0.027 ± 0.002 mg kg^−1^	108.00 ± 0.08
	0.087 mg kg^−1^	0.089 ± 0.002 mg kg^−1^	101.41 ± 2.58
	0.125 mg kg^−1^	0.135 ± 0.002 mg kg^−1^	108.05 ± 1.22

**Table 2:** Comparison of the method for Cd^2+^ detection.

Methods	Materials	LOD	Real Samples	Reference
Colorimetric	Cellulose nano-whiskers, AuNPs	60 nM	Water	[27]
Colorimetric	Aptamer, AuNPs	14.56 nM	Water	[28]
Colorimetric	Aptamer, AuNP, MoS_2_	9.1 nM	White wine	[3]
Fluorescence	Aptamer, SYBR^®^ green Ι	4.42 nM	Water	[29]
Fluorescence	Aptamer, FAM	2.15 nM	Water	[11]
Phosphorescence	Aptamer, Zn_2_GeO_4_:Mn, BHQ_1_	0.52 nM	Water, Clams	This work

Abbreviations: Cd^2+^: Cadmium ions; LOD: Limit of detection; AuNPs: Gold nanoparticles; MoS_2_: Molybdenum disulfide; FAM: Carboxyfluorescein; Zn_2_GeO_4_:Mn: Manganese doped zinc germanate; BHQ_1_: Black hole quencher 1.

## Supplementary Materials

The following are available online at https://www.mdpi.com/2304-8158/9/12/1758/s1, Figure S1: The corresponding length distribution of Zn_2_GeO_4_:Mn synthesized at 2, 4, 6, 8 h (A–D); Width distribution of Zn_2_GeO_4_:Mn synthesized at 2, 4, 6, 8 h (E–H), Figure S2: EDS elemental mapping of Zn_2_GeO_4_:Mn synthesized at 2 h (A), 4 h (B), 6 h (C), 8 h (D). High-resolution Zn 2p XPS spectra of Zn_2_GeO_4_:Mn synthesized at 2 h (E), 4 h (F), 6 h (G), 8 h (H), Figure S3: High-resolution Ge 2p XPS spectra of Zn_2_GeO_4_:Mn synthesized at 2 h (E), 4 h (F), 6 h (G), 8 h (H). High-resolution O_1s_ XPS spectra of Zn_2_GeO_4_:Mn synthesized at 2 h (E), 4 h (F), 6 h (G), 8 h (H), Table S1: The sequences of biotin labelled Cd^2+^-binding aptamer (ssDNA), and its black hole quencher 1 (BHQ_1_) labelled complementary strand, Table S2: The quantum yield (QY) Zn_2_GeO_4_:Mn synthesized at 2, 4, 6, 8 h.
