# Supplementary material for: A Highly Sensitive “on-off” Time-Resolved Phosphorescence Sensor Based on Aptamer Functionalized Magnetite Nanoparticles for Cadmium Detection in Food Samples
Source: Foods. 2020 Nov 27;9(12):1758. doi: 10.3390/foods9121758 (PMC7760395; doi:10.3390/foods9121758)
Supplement: Supplementary file 1 [file foods-09-01758-s001.pdf]

Supporting information

A highly sensitive "on-off" time-resolved phosphorescence sensor based on aptamer functionalized magnetite nanoparticles for cadmium detection in food samples

Bin Lai [1,2,3], Ruiying Wang [1,2,3], Xiaoting Yu [1,2,3], Haitao Wang [1,2,3], Zhouping Wang [4] and Mingqian Tan [1,2,3,*]

1. School of Food Science and Technology, Dalian Polytechnic University, Qinggongyuan 1, Ganjingzi District, Dalian 116034, China; bin.lai33@gmail.com (B.L.); wangruiying2020_0@163.com (R.W.); xiaotingddpp12@163.com (X.Y.); wanght@dlpu.edu.cn (H.W.)
2. National Engineering Research Center of Seafood, Dalian Polytechnic University, Dalian 116034, China
3. Collaborative Innovation Center of Seafood Deep Processing, Dalian Polytechnic University, Dalian 116034, China
4. School of Food Science and Technology, Jiangnan University, Wuxi 214122, China; wangzp@jiangnan.edu.cn

* Correspondence: mqtan@dlpu.edu.cn; +86-411-86318657

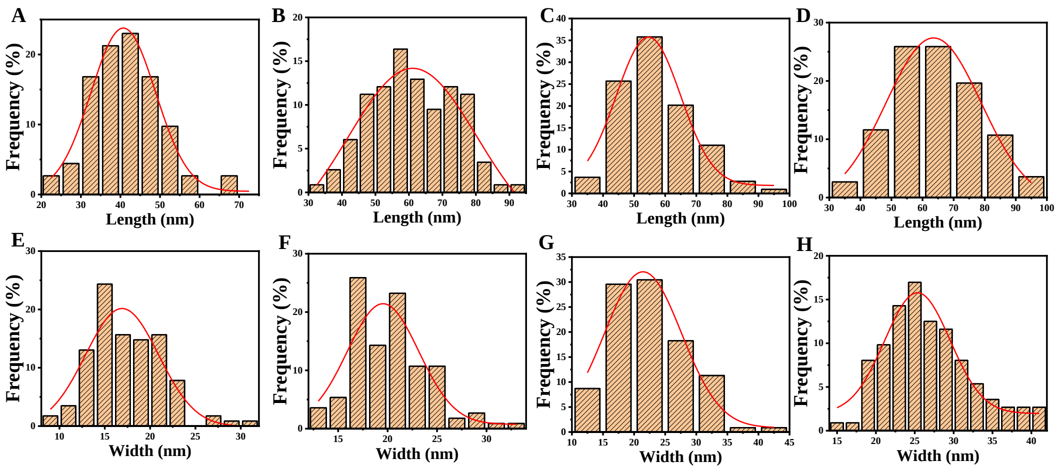

Figure S1. Length distribution of Zn_2GeO_4:Mn synthesized at 2, 4, 6, 8 h (A-D); Width distribution of Zn_2GeO_4:Mn synthesized at 2, 4, 6, 8 h (E-H).

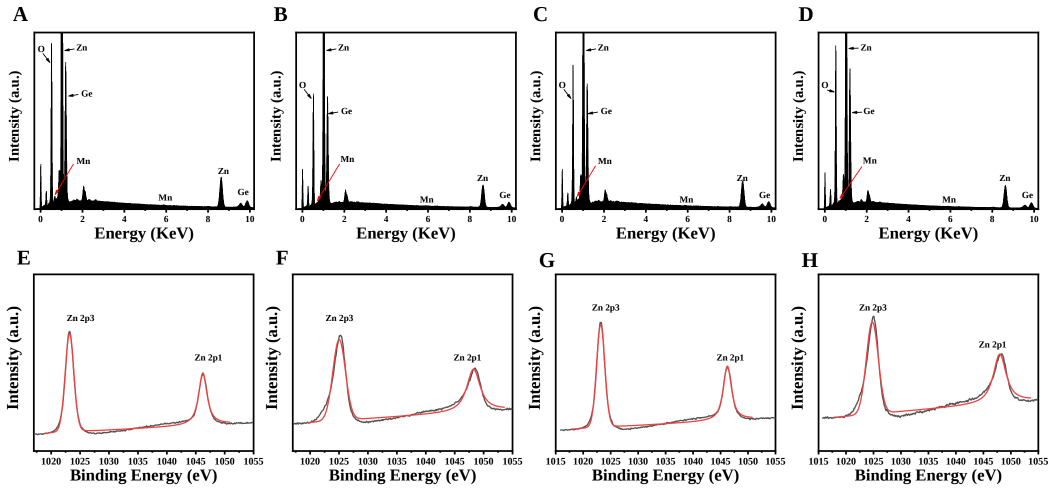

Figure S2. EDS elemental mapping of Zn_2GeO_4:Mn synthesized at 2 h (A), 4 h (B), 6 h (C), 8 h (D). High-resolution Zn_{2p} XPS spectra of Zn_2GeO_4:Mn synthesized at 2 h (E), 4 h (F), 6 h (G), 8 h (H).

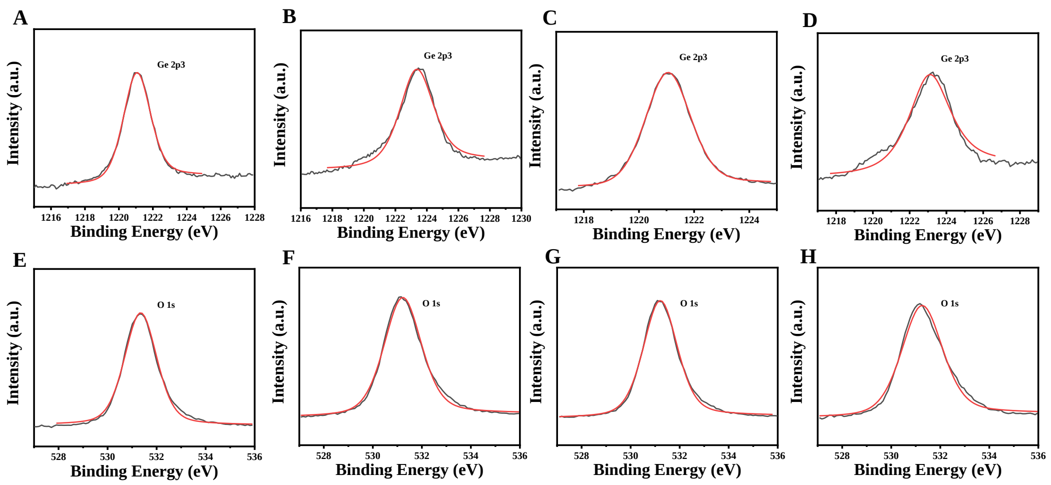

Figure S3. High-resolution Ge_{2p} XPS spectra of Zn_2GeO_4:Mn synthesized at 2 h (E), 4 h (F), 6 h (G), 8 h (H). High-resolution O_{1s} XPS spectra of Zn_2GeO_4:Mn synthesized at 2 h (E), 4 h (F), 6 h (G), 8 h (H).

Table S1. The sequences of biotin labelled Cd^{2+}-binding aptamer (ssDNA), and its black hole quencher 1 (BHQ_1) labelled complementary strand.

Sequence	
biotin labelled Cd^{2+}-binding aptamer	5′-Biotin-ACCGACCGTGCTGGACTCTGGACTGTTGTGGTATTATTTTTGGTTGTGCAGTATGAGCGAGCGTTGCG-3′
black hole quencher 1 (BHQ_1) labelled complementary strand	5′-BHQ_1-CGCAACGCTCGCTCATACTGCACAACCAAA-3′

Table S2. The quantum yield (QY) Zn_2GeO_4:Mn synthesized at 2, 4, 6, 8 h.

	2 h	4 h	6 h	8 h
Quantum yields (%)	8.42	12.22	14.18	17.82
